# ﻿A newly discovered species of the genus *Scorpiops* Peters, 1861, subgenus *Euscorpiops* Vachon, 1980 from Doi Phu Kha National Park, Thailand (Scorpiones, Scorpiopidae)

**DOI:** 10.3897/zookeys.1241.142549

**Published:** 2025-06-12

**Authors:** Eric Ythier, Ondřej Košulič, Wasin Nawanetiwong, Wilson R. Lourenço

**Affiliations:** 1 BYG Taxa, 382 rue des Guillates, 71570 Romanèche-Thorins, France BYG Taxa Romanèche-Thorins France; 2 Department of Forest Protection and Wildlife Management, Faculty of Forestry and Wood Technology, Mendel University in Brno, Zemědělská 3, Brno, Czech Republic Mendel University in Brno Brno Czech Republic; 3 Department of Biology, Faculty of Science, Chulalongkorn University, Bangkok, 103303, Thailand Chulalongkorn University Bangkok Thailand; 4 Muséum national d’Histoire naturelle, Sorbonne Universités, Institut de Systématique, Evolution, Biodiversité (ISYEB), UMR7205-CNRS, MNHN, UPMC, EPHE, CP 53, 57 rue Cuvier, 75005 Paris, France Muséum national d’Histoire naturelle, Sorbonne Universités Paris France

**Keywords:** Description, ecology, forest, morphology, scorpion, Southeast Asia, taxonomy

## Abstract

A new scorpion species, Scorpiops (Euscorpiops) doiphukha**sp. nov.**, belonging to the family Scorpiopidae Kraepelin, 1905, is described based on 12 specimens of both sexes (three adults and nine immatures) collected in Doi Phu Kha National Park, Nan Province, Thailand. The new species presents key features exhibited by scorpions of the subgenus Euscorpiops and can be characterized notably by a large size, a sexual dimorphism strongly marked with male pedipalps elongated, a distinct trichobothrial pattern and other morphological features. This new taxon represents the 115^th^ species among the currently recognized species for the genus *Scorpiops* Peters, 1861, and the 44^th^ species described for the subgenus Euscorpiops Vachon, 1980. It is likely an endemic element of Thailand’s scorpion fauna, raising the number of known Scorpiops (Euscorpiops) species in the country to 13. Ecological and distributional aspects of the new species are discussed and compared with closely related *Scorpiops* species, highlighting its distinctiveness within the genus.

## ﻿Introduction

As previously commented (e.g., Lourenço and Košulič 2018; Lourenço 2019; Lourenço and Ythier 2022; Nawanetiwong et al. 2024), the generic composition of the family Scorpiopidae Kraepelin, 1905 has been widely accepted since the early 2000s. This classification recognized nine genera: *Scorpiops* Peters, 1861; *Parascorpiops* Banks, 1928; *Dasyscorpiops* Vachon, 1974; *Alloscorpiops* Vachon, 1980; *Euscorpiops* Vachon, 1980; *Neoscorpiops* Vachon, 1980; *Laoscorpiops* Lourenço, 2013; *Vietscorpiops* Lourenço & Pham, 2015; and *Plethoscorpiops* Lourenço, 2017. In a subsequent revision of the family, Kovařík et al. (2020) synonymized all genera within Scorpiopidae with *Scorpiops*, except for *Parascorpiops* which remained a valid genus. Later, Lourenço and Ythier (2022) proposed revalidating *Dasyscorpiops*, *Alloscorpiops*, *Euscorpiops*, *Neoscorpiops*, and *Plethoscorpiops* as subgenera of *Scorpiops*, while accepting the synonymization of *Laoscorpiops* and *Vietscorpiops* and maintaining *Parascorpiops* as an independent genus.

In the present study, a new species belonging to the subgenus Euscorpiops is described from Doi Phu Kha National Park located in the Nan Province of northeastern Thailand. *Scorpiopsdoiphukha* sp. nov. represents the 44^th^ species of the subgenus Euscorpiops (the 13^th^ described from Thailand) and the total number of *Scorpiops* species is now raised to 115. This new taxon may represent one endemic element for the scorpion fauna of Thailand.

## ﻿Methods

Illustrations and measurements were produced using a Motic SMZ-171 stereomicroscope with an ocular micrometer. Habitus photographs were taken with a Canon EOS RP. Map was made using Google Maps. Habitus photographs and maps were edited in Adobe Photoshop. Measurements follow Stahnke (1970) and are given in millimeters. Trichobothrial notations follow Vachon (1974), and morphological terminology mostly follows Vachon (1952) and Hjelle (1990). A collecting permit was provided by the Department of National Parks, Wildlife and Plant Conservation, Ministry of Natural Resources and Environment in Thailand.

## ﻿Taxonomic treatment

### ﻿Family Scorpiopidae Kraepelin, 1905

**Genus *Scorpiops* Peters, 1861**

#### Subgenus ﻿ Euscorpiops

Taxon classificationAnimaliaScorpionesScorpiopidae

Vachon, 1980

8E11A9B3-708E-50CE-8F86-A060143E9276

##### Composition of the subgenus Euscorpiops.

- S. (E.) alexandreanneorum (Lourenço, 2013) (Laos)
- S. (E.) artemisae (Kovařík, Košulič, Šťáhlavský, Dongkhamfu & Wongprom, 2015) (Myanmar)
- S. (E.) asthenurus Pocock, 1900 (Bhutan, India)
- S. (E.) bahunetra Deshpande, Joshi, Ukale, Bastawade, Tang, Gowande, Monod & Sulakhe, 2025 (India)
- S. (E.) beccaloniae (Kovarík, 2005) (Myanmar)
- S. (E.) bhutanensis (Tikader & Bastawade, 1983) (Bhutan)
- S. (E.) binghamii Pocock, 1893 (Myanmar, Thailand)
- S. (E.) cavernicola (Lourenço & Pham, 2013) (Vietnam)
- S. (E.) chiangmai (Lourenço, 2019) (Thailand)
- S. (E.) ciki Kovařík, Lowe, Stockmann & Šťáhlavský, 2020 (Myanmar)
- S. (E.) dakrong (Lourenço & Pham, 2014) (Vietnam)
- S. (E.) deshpandei Tang, Ouyang, Liu & Šťáhlavský, 2024 (China)
- S. (E.) dii Kovařík, Lowe, Stockmann & Šťáhlavský, 2020 (Thailand)
- S. (E.) doiphukha sp. nov. (Thailand)
- S. (E.) dunlopi Kovařík, Lowe, Stockmann & Šťáhlavský, 2020 (Thailand)
- S. (E.) kaftani (Kovarík, 1993) (Vietnam)
- S. (E.) kamengensis (Bastawade, 2006) (India)
- S. (E.) krachan Nawanetiwong, Košulič, Warrit, Lourenço & Ythier, 2024 (Thailand)
- S. (E.) kubani (Kovarík, 2004) (Laos)
- S. (E.) lii (Di & Qiao, 2020) (China)
- S. (E.) longimanus (Pocock, 1893) (Bangladesh, India, Myanmar)
- S. (E.) lowei Tang, 2022 (China)
- S. (E.) montanus (Karsch, 1879) (India, Pakistan)
- S. (E.) neradi (Kovařík, Pliskova & Šťáhlavský, 2013) (Thailand)
- S. (E.) novaki (Kovařík, 2005) (China)
- S. (E.) orioni (Kovařík, Košulič, Šťáhlavský, Dongkhamfu & Wongprom, 2015) (Thailand)
- S. (E.) phatoensis Kovařík, Lowe, Stockmann & Šťáhlavský, 2020 (Thailand)
- S. (E.) piceus Lourenço & Ythier, 2022 (Laos)
- S. (E.) prasiti Kovařík, Lowe, Stockmann & Št’áhlavský, 2020 (Thailand)
- S. (E.) problematicus (Kovařík, 2000) (Thailand)
- S. (E.) puerensis (Di, Wu, Cao, Xiao & Li, 2010) (China)
- S. (E.) reini Tang, 2024 (China)
- S. (E.) schumacheri Kovařík, Lowe, Stockmann & Šťáhlavský, 2020 (Thailand)
- S. (E.) sejnai (Kovařík, 2000) (Vietnam)
- S. (E.) sherwoodae Kovařík, Lowe, Stockmann & Šťáhlavský, 2020 (Thailand)
- S. (E.) shidian (Qi, Zhu & Lourenço, 2005) (China)
- S. (E.) solegladi Kovařík, Lowe, Stockmann & Šťáhlavský, 2020 (Vietnam)
- S. (E.) tangae Kovařík, Šťáhlavský & Stockmann, 2024 (Laos)
- S. (E.) thaomischorum (Kovarík, 2012) (Vietnam)
- S. (E.) tongtongi Tang, 2022 (China)
- S. (E.) vachoni (Qi, Zhu & Lourenço, 2005) (China)
- S. (E.) yangi (Zhu, Zhang & Lourenço, 2007) (China)
- S. (E.) xui (Sun & Zhu, 2010) (China)
- S. (E.) zhangshuyuani (Ythier, 2019) (China)

#### ﻿ Scorpiops (Euscorpiops) doiphukha sp. nov.

Taxon classificationAnimaliaScorpionesScorpiopidae

CFE9A601-AE49-516A-83AC-2B30A63B5798

https://zoobank.org/8AEC26C8-4236-4EA9-B287-AA2121192CB5

Figs 1 , 2 , 3 , 4

##### Type material.

Thailand • Nan Province, Doi Phu Kha National Park, GPS 19°11'53"N, 101°5'6"E, ca. 1400 m a.s.l., hill evergreen forest, 28/V/2024 (O. Košulič leg.). • Adult male holotype, 2 adult females (one incomplete), 2 pre-adult males, 3 juvenile females and 4 juvenile males paratypes. Adult male holotype, one adult female (complete) paratype and one pre-adult male paratype are deposited in Muséum national d’Histoire naturelle, Paris, France. Other paratypes are deposited at Department of Biology, Faculty of Science, Chulalongkorn University, Bangkok, Thailand.

**Figure 1.**
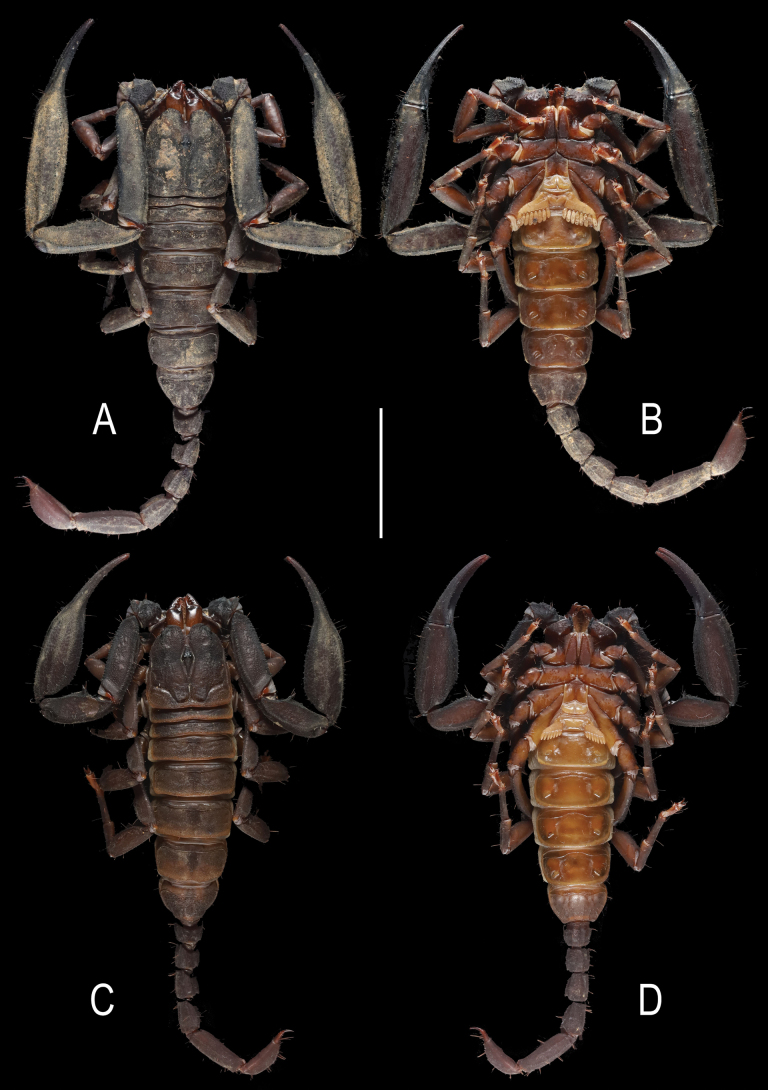
Scorpiops (Euscorpiops) doiphukha sp. nov. **A, B.** Male holotype, habitus, dorsal and ventral aspects, respectively; **C, D.** Female paratype, habitus, dorsal and ventral aspects, respectively. Scale bar: 2 cm.

##### Etymology.

The specific name refers to the National Park of Doi Phu Kha where the new species was collected.

**Figure 2.**
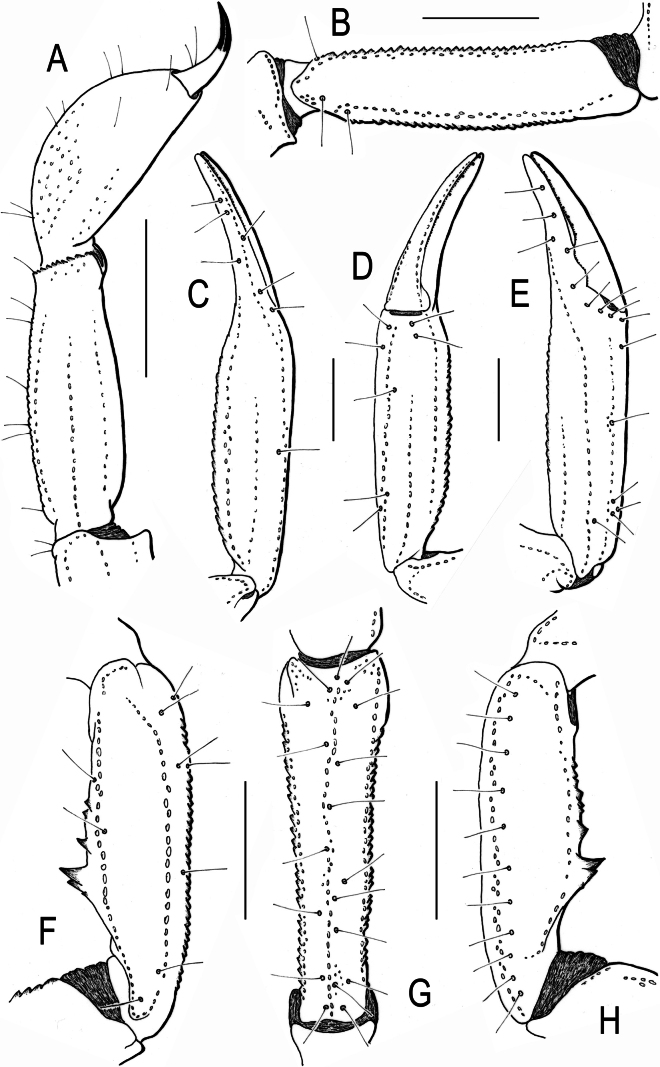
Scorpiops (Euscorpiops) doiphukha sp. nov. Male holotype, trichobotrial pattern. **A.** Metasomal segment V and telson, lateral aspect; **B.** Femur, dorsal aspect; **C–E.** Chela; **C.** Dorsal, ventral, and external aspects, respectively; **F–H.** Patella, dorsal, external, and ventral aspects, respectively. Scale bars: 5 mm.

##### Diagnosis.

The new species exhibits the general characteristics of the subgenus Euscorpiops (Vachon 1980; Soleglad and Sissom 2001). Male holotype and female paratype with respectively 70.3 and 63.3 mm in total length, defining the new species as large in relation to most other species of the genus. General coloration yellowish brown (female) to reddish brown (male). Pectines with 8–8 teeth in male, 7–7 teeth in female; two marginal and one middle lamellae present; fulcra present in adult specimens. Annular ring conspicuous in both sexes; telson length/depth ratio 2.92 in male, 3.82 in female. Sexual dimorphism strongly marked with male pedipalps markedly elongated; chela length/width ratio 5.35 in male, 3.95 in female. Chela fingers undulate in both sexes; movable fingers with two parallel longitudinal rows of granules almost fused, formed by a row of about 97 median granules and a row of about 72 inner accessory granules; 3–4 inner granules and 13–15 outer granules are also present. Trichobothriotaxy of type C (Vachon 1974, 1980); three trichobothria on femur (dorsal, internal, and external); patella with 2 dorsal, 1 internal, 11–12 ventral and 18 external trichobothria; chelal manus with 4 ventral, 2 dorsal (*Dt*, *Db*), 2 internal (*ib*, *it*), 1 *Est*, 5 *Et*, 1 *Esb* and 3 trichobothria in the *Eb* series; trichobothrium *Eb_3_* located in distal half of manus, between trichobothria *Dt* and *Est*.

**Figure 3.**
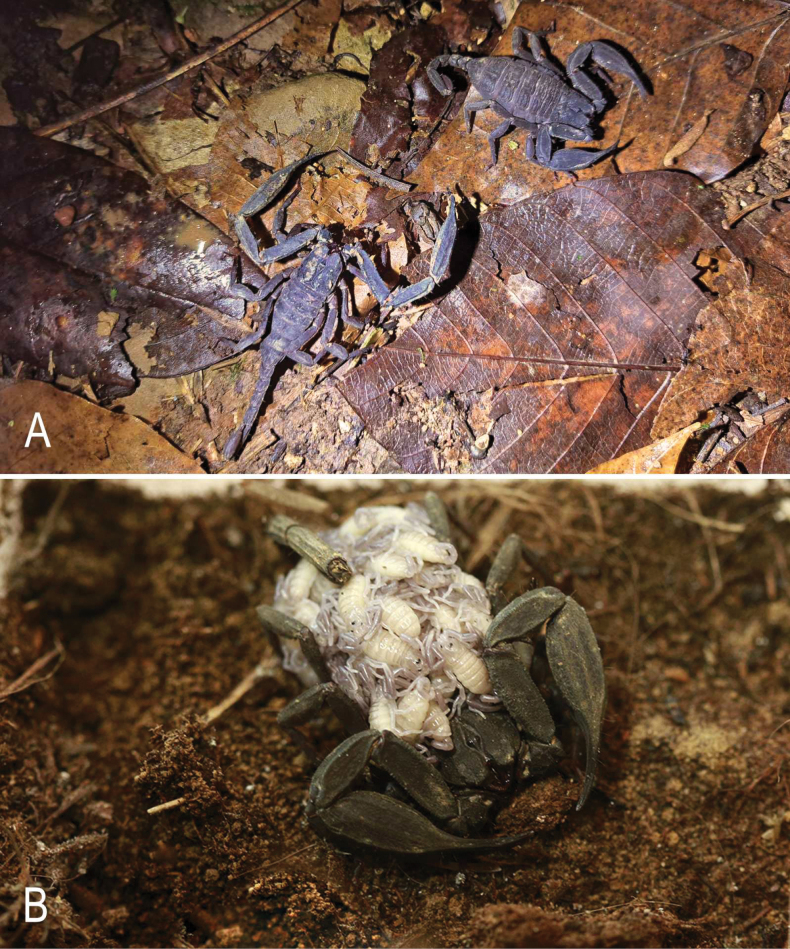
Scorpiops (Euscorpiops) doiphukha sp. nov., alive. **A.** Male holotype and female paratype, in their natural habitat; **B.** Female, alive with pre-juveniles (instar I).

##### Description.

Based on male holotype and one female paratype.

##### Coloration (in alcohol).

Basically, yellowish brown (female) to reddish brown (male). Carapace yellowish brown (female) to reddish brown (male) with paler zones posteriorly and on furrows. Tergites yellowish (female) to yellowish brown (male) with darker pigmentation anteriorly. Metasomal yellowish brown, darker in male; telson yellowish brown; base of aculeus yellowish and tip reddish. Chelicerae yellowish with conspicuous variegated brownish spot; fingers brownish with reddish teeth. Pedipalps yellowish brown (female) to reddish brown (male); fingers darker than chela manus, reddish brown (female) to almost blackish (male). Legs yellowish brown, intensely spotted with brownish. Venter, coxapophysis and sternum brownish yellow; sternites yellowish with the VII darker; genital operculum and pectines pale yellow.

##### Morphology.

Carapace moderately granular; furrows moderately deep. Median eyes anterior to the middle of carapace; three pairs of lateral eyes, the third pair only slightly smaller than the first two. Sternum pentagonal, slightly longer than wide. Tergites moderately granular; VII with five carinae, moderately marked; median carinae vestigial. Pectines large in male and reduced in female with a pectinal tooth count of 8–8 and 7–7, respectively; two marginal and one middle lamellae present; fulcra present in adult specimens, absent immatures. Sternites smooth, with oval spiracles; sternite VII with four vestigial carinae and some minute granulations. Metasomal segments I to V with 10-8-8-8-7 carinae; dorsal carinae on segments II to IV with several spinoid granules and one larger posterior spinoid granule; metasomal tegument moderately granulated; ventral carina on segment V without spinoid granules. Telson vesicle almost smooth, only some minute granulation laterally; annular ring conspicuous in both sexes; telson length/depth ratio 2.92 in male, 3.82 in female. Setation weak on metasomal segments and telson. Pedipalps: femur with dorsal internal, dorsal external, ventral internal and ventral external carinae strongly marked; tegument moderately granular. Patella with dorsal internal, dorsal external, ventral internal, ventral external and external carinae strongly marked; two strongly marked spinoid granules present on internal aspect, ventral bigger than dorsal; tegument moderately granular. Chela with dorsal marginal, external secondary, ventral internal and ventral carinae strongly marked; other carinae moderate; tegument moderately granulated. Sexual dimorphism strongly marked with male pedipalps markedly elongated; chela length/width ratio 5.35 in male, 3.95 in female. Chelal fingers undulate in both sexes; movable fingers with two parallel longitudinal rows of granules almost fused, formed by a row of about 97 median granules and a row of about 72 inner accessory granules; 3–4 inner granules and 13–15 outer granules are also present. Cheliceral dentition as defined for the family (Vachon 1963); 4–5 teeth on ventro-internal face of movable finger. Trichobothriotaxy of type C (Vachon 1974, 1980); three trichobothria on femur (dorsal, internal and external); patella with 2 dorsal, 1 internal, 11–12 ventral and 18 external trichobothria; chelal manus with 4 ventral, 2 dorsal (*Dt*, *Db*), 2 internal (*ib*, *it*), 1 *Est*, 5 *Et*, 1 *Esb* and 3 trichobothria in the *Eb* series. Trichobothrium *Eb_3_* distal in relation to *Eb_2_* (Vachon 1974, 1980), located in distal half of manus, between trichobothria *Dt* and *Est*. Legs tarsi with 4–5 long setae on lateral and dorsal surfaces and 5–8 ventral spines; tibial spurs absent.

##### Morphometric values.

Adult male holotype and adult female paratype of Scorpiops (Euscorpiops) doiphukha sp. nov. Total length including the telson 70.33/63.29. Carapace: length 13.13/11.13; anterior width 6.13/5.25; posterior width 10.75/10.00. Mesosoma length 25.38/24.63. Metasomal segments. I: length 3.13/2.63, width 3.63/3.00; II: length 3.38/2.88, width 3.38/2.88; III: length 3.63/3.38, width 3.13/2.88; IV: length 4.38/3.88, width 3.00/2.75; V: length, 8.25/7.13, width 3.00/2.75, depth 3.00/2.50. Telson length 8.75/7.63; vesicle: width 2.75/2.00, depth 3.00/2.00. Pedipalp: femur length 15.13/10.63, width 3.63/3.63; patella length 12.75/8.88, width 3.63/3.75; chela length 26.75/20.75, width 5.00/5.25, depth 4.38/5.13. Movable finger length 11.50/10.25.

##### Relationships.

**Figure 4.**
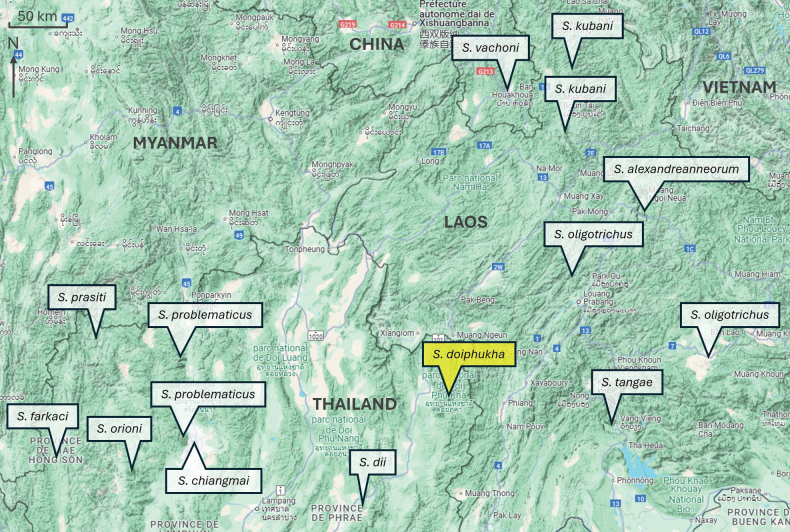
Map showing the type locality of Scorpiops (Euscorpiops) doiphukha sp. nov. and the distribution of the geographically closest *Scorpiops* species discussed in this work.

In respect to several characters, the most similar species seem to be S. (E.) dii, S. (E.) orioni, and S. (E.) prasiti, described from the Thailand Provinces of Phrae, Chiang Mai, and Mae Hong Son, respectively (Fig. 4). *Scorpiopsdoiphukha* sp. nov. can however be separated from these species notably by the following main features:

- larger size with 70.3 mm in male (38 mm in *S.dii*, 49 mm in *S.orioni*, 52 mm in *S.prasiti*) and 63.3 mm in female (58 mm in *S.dii*, 58 mm in *S.orioni*, 53 mm in *S.prasiti*) with male larger than female (female larger than or equivalent to male in all three species);
- male telson deeper with length to depth ratio 2.92 (3.26 in *S.dii*, 3.4 in *S.orioni* and *S.prasiti*);
- pedipalp chela slenderer than in *S.dii* and *S.orioni* with length to width ratio 5.35 in male (3.10 in *S.dii*, 4.60 in *S.orioni*) and 3.95 in female (3.17 in *S.dii*, 3.0–3.4 in *S.orioni*);
- pedipalp chela movable fingers with about 72 inner accessory granules (50–56 in *S.dii*) and 3–4 inner granules (6 in *S.dii* and *S.orioni*, 7 in *S.prasiti*);
- pedipalp patella with 11–12 ventral and 18 external trichobotria (14–15 and 20–22 respectively in *S.prasiti*).

The new species can also be distinguished from the other geographically closest species (Fig. 4), notably by the following main features:

- S. (E.) tangae, described from Vientiane Province, Laos: smaller, with 33 mm in male; male pedipalp chela wider with length to width ratio 3.7; pedipalp chela movable fingers with about 73 median granules and 45 inner accessory granules.
- S. (E.) chiangmai, described from Chiang Mai Province, Thailand: pedipalp patella with 17 ventral and 16 external trichobotria.
- S. (E.) problematicus, described from Chiang Mai Province, Thailand: smaller, with 46 mm in male and 47 mm in female; male telson thinner with length to depth ratio 3.3; pedipalp chela wider with length to width ratio 3–3.33 in both sexes; pedipalp chela movable fingers with about 75 median granules and 5–6 inner granules.
- S. (E.) alexandreanneorum, described from Luang Prabang Province, Laos: smaller, with 38 mm in both sexes; male telson thinner with length to depth ratio 3.9; male pedipalp chela slenderer with length to width ratio 6.8; male pedipalp fingers straight; pedipalp chela movable fingers with about 70 median granules and 8 outer granules; pedipalp patella with 14 ventral and 21 external trichobotria.
- S. (E.) kubani, described from Phongsaly Province, Laos: smaller, with 39 mm in male and 44 mm in female; male telson thinner with length to depth ratio 3.5; pedipalp chela wider with length to width ratio 3.1–3.2 in both sexes; pedipalp chela movable fingers with about 70 median granules and 50 inner accessory granules.
- S. (E.) vachoni, described from Yunnan Province, China: smaller, with 47–54 mm in male and 42–55 mm in female; pedipalp chela wider with length to width ratio 2.8–3.2 in both sexes; pedipalp chela movable fingers with 6–8 inner granules.
- S. (S.) oligotrichus, from Luang Prabang and Xiangkhouang Provinces: smaller, with 32–50 mm in both sexes; male telson thinner with length to depth ratio 3.3–3.5; male pedipalp chela wider with length to width ratio 3.2; female pedipalp fingers straight; pedipalp chela movable fingers with about 55 median granules, about 40 inner accessory granules and 9 outer granules; pedipalp patella with 9 ventral trichobotria; trichobothrium *Eb _3_* located in proximal half of manus, between trichobothria *Dt* and *Db*.
- S. (S.) farkaci, described from Mae Hong Son Province, Thailand: smaller, with 25–33 mm in male and 27–37 mm in female; female telson deeper with length to depth ratio 2.8; pedipalp chela wider with length to width ratio 2.7–3.1 in male and 2.4–25 in female; female pedipalp fingers straight; pedipalp chela movable fingers with about 55 median granules and 25–30 inner accessory granules; pedipalp patella with 9 ventral trichobotria; trichobothrium *Eb _3_* located in proximal half of manus, between trichobothria *Dt* and *Db*.

### ﻿Ecology and distribution of *Scorpiopsdoiphukha* sp. nov.

**Figure 5.**
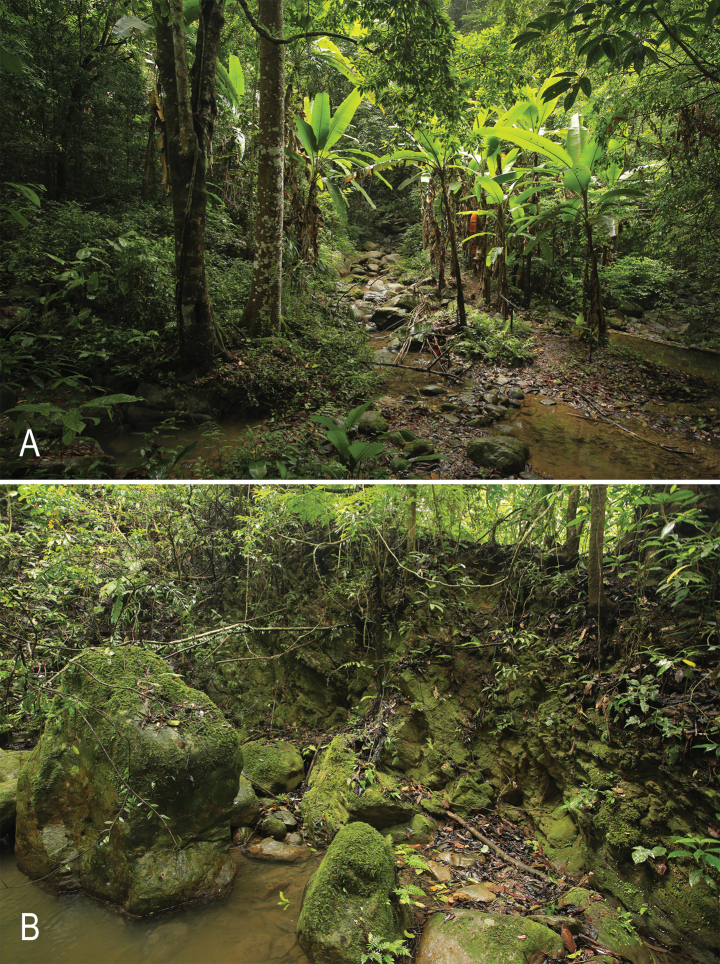
Natural habitat of Scorpiops (Euscorpiops) doiphukha sp. nov. in Doi Phu Kha National Park, Nan Province, Thailand. **A.** Track trail near a small stream; **B.** Rock wall where the male holotype and female paratype were discovered. Both specimens were found hiding in small rock crevices.

Scorpiops (Euscorpiops) doiphukha sp. nov. is endemic to the hill evergreen forests of Doi Phu Kha National Park in Nan Province, Thailand, at approximately 1500 m a.s.l. These forests are characterized by dense vegetation, cooler temperatures (especially during dry season), and high humidity, creating an ideal microhabitat for the species (Fig. 5A). The scorpions were primarily observed hiding within rock crevices on rock walls and were exclusively found at night in ambush positions, waiting for potential prey to crawl near their hiding spots (Fig. 5B). Juvenile specimens were often found outside these crevices, crawling on rock and soil walls, frequently near the refuges of adult individuals. The co-occurrence of juvenile individuals with adults suggests a localized reproductive strategy, potentially synchronized with the seasonal climatic patterns of this tropical montane environment.

Compared to most other *Scorpiops* species occurring in Thailand, *S.doiphukha* sp. nov. occupies a high-altitude ecosystem that is relatively undisturbed. Other species such as S. (E.) dii, S. (E.) prasiti, and S. (E.) orioni, which are also found in northern Thailand, inhabit similar forest environments but at varying altitudes and exhibit slightly different ecological niches (Kovařík et al. 2015). For instance, S. (E.) prasiti and S. (E.) orioni are known from evergreen and mixed forests at lower elevations, where temperatures are warmer and seasonal variability may be less pronounced compared to the higher-altitude forests of Doi Phu Kha.

However, the ecological differences in habitat preference are especially emphasized when comparing *S.doiphukha* sp. nov. to the recently described *S.krachan* (Nawanetiwong et al. 2024), a southern species found at low elevations in Kaeng Krachan National Park. Although both species inhabit evergreen forests, their habitats are not identical. The differences appear to align with variations in seasonal dynamics. At Doi Phu Kha National Park, the seasons vary annually, encompassing rainy, warm and winter periods. In contrast, Kaeng Krachan National Park experiences relatively stable weather throughout the year, with a pronounced rainy season. The average annual precipitation there is 986–1,140 mm, resulting in consistently high humidity. These differences in seasonal variability may drive local scorpion populations to adapt to their specific environments to survive. This process likely contributes to the distinct evolutionary trajectories of these species, leading to speciation. The comparison between the northern *S.doiphukha* sp. nov. and the southern *S.krachan* suggests that *S.doiphukha* sp. nov. exhibits a greater tolerance to seasonal variability than *S.krachan*. Furthermore, this highlights differences between the species that extend beyond morphology, emphasizing the influence of ecological and environmental factors in shaping their divergence.

In surrounding countries, species such as S. (E.) alexandreanneorum in Laos and S. (E.) vachoni in China similarly inhabit forested environments but tend to occupy lower altitudes or different climatic zones (Lourenço 2013). These variations highlight the adaptability of *Scorpiops* species to diverse ecological conditions while also emphasizing the specialized habitat requirements of *S.doiphukha* sp. nov. Unlike its relatives, the new species seems restricted to the montane forests of Doi Phu Kha, suggesting a strong dependency on the unique conditions of this high-altitude habitat. This specialization may be an adaptation to the cooler, wetter microclimate and the potentially reduced competition found at higher elevations.

Its presence in a protected area like Doi Phu Kha National Park emphasizes the importance of conserving such habitats, as they support not only endemic species like *S.doiphukha* sp. nov. but also a broader spectrum of biodiversity (Trisurat et al. 2010). This is particularly significant given the persistent threat of deforestation in parts of the park, driven by efforts to create agricultural terraces for crops such as corn (Turkelboom et al. 2008). Such activities can lead to habitat degradation and fragmentation, posing risks to the survival of species uniquely adapted to these environments. In this context, *S.doiphukha* sp. nov. can serve as an important flagship species for the conservation of local ecosystems, symbolizing the need to balance agricultural practices with environmental preservation. Furthermore, the ecological characteristics of *S.doiphukha* sp. nov. reflect its specialization for high-altitude habitats like hill evergreen forests, which distinguishes it from other *Scorpiops* species that often occupy lower-altitude or significantly disturbed environments across Southeast Asia. This narrow habitat preference not only highlights the species’ ecological significance but also underscores the urgent need for targeted conservation measures to protect the unique biodiversity of Doi Phu Kha National Park.

## Supplementary Material

XML Treatment for Euscorpiops

XML Treatment for Scorpiops (Euscorpiops) doiphukha

## ﻿Additional information

### Author ORCIDs

Eric Ythier https://orcid.org/0000-0002-3194-5184

Ondřej Košulič https://orcid.org/0000-0003-2199-1694

Wilson R. Lourenço https://orcid.org/0000-0002-2386-363X

### Data availability

All of the data that support the findings of this study are available in the main text.
